# Heterotopic Triplet Pregnancy After Clomiphene Citrate

**DOI:** 10.31486/toj.20.0150

**Published:** 2021

**Authors:** James J. Morong, Jennifer Janssen, Jessica C. Morgan, Sophia M. Rodriguez

**Affiliations:** ^1^ Department of Obstetrics and Gynecology, University of Illinois at Chicago, Chicago, IL; ^2^ Department of Obstetrics and Gynecology, Advocate Christ Medical Center, Oak Lawn, IL

**Keywords:** *Pregnancy–heterotopic*, *reproductive techniques–assisted*, *superfetation*

## Abstract

**Background:** Heterotopic pregnancy is an exceedingly rare condition in which an intrauterine and extrauterine pregnancy coexist. Superfetation refers to the coexistence of 2 or more fetuses of different gestational ages as a result of ovulation, fertilization, and implantation during an ongoing pregnancy. We present a case of heterotopic triplet pregnancy with a difference in gestational age by crown rump length of more than 1 week between the twin intrauterine pregnancy and the singleton tubal ectopic.

**Case Report:** A 31-year-old gravida 3, para 2002 presented to the emergency department with abdominal pain at 9 weeks 2 days’ gestation dated by last menstrual period, consistent with ultrasound. She was discharged home with a diagnosis of ruptured hemorrhagic cyst but returned 4 days later with ruptured tubal ectopic pregnancy measuring 9 weeks’ gestation and ongoing twin gestation measuring 10 weeks 1 day. She was taken to the operating room for laparoscopic salpingectomy, and ectopic pregnancy was confirmed on tissue diagnosis.

**Conclusion:** Heterotopic pregnancy presents a diagnostic challenge for obstetricians/gynecologists. Superfetation has never been demonstrably proven in humans but has been suggested in the literature. This report adds to the literature that perhaps superfetation can be artificially induced in humans in the presence of assisted reproductive technologies.

## INTRODUCTION

Heterotopic pregnancy is an exceedingly rare condition in which an intrauterine and extrauterine pregnancy coexist. Heterotopic pregnancy can happen spontaneously but is more common in the setting of assisted reproductive technologies (ART) such as the use of estrogen modulators that cause superovulation–the release of multiple oocytes capable of fertilization.^1^ Apart from ART, risk factors for heterotopic pregnancy include pelvic inflammatory disease, adhesions, history of ectopic pregnancy, and ovarian hyperstimulation syndrome. Because of the rarity of heterotopic pregnancy, the diagnosis is often missed or delayed.^2^ A visualized adnexal mass in the context of intrauterine pregnancy is frequently presumed to be a corpus luteum or hemorrhagic cyst, and any free fluid or abdominal pain can be attributed to those diagnoses. Similarly, the doubling of beta human chorionic gonadotropin (ß-hCG) in a 48-hour period (used to distinguish a viable intrauterine pregnancy from an abnormal pregnancy) does not serve as a useful diagnostic tool when intrauterine and extrauterine pregnancies coexist.^3^

Superfetation refers to the coexistence of 2 or more fetuses of different gestational ages as a result of ovulation, fertilization, and implantation during an existing pregnancy.^4^ Superfetation is purported in certain mammalian species such as rodents and rabbits, marsupials, and other species that exhibit placentotrophy. The phenomenon has been demonstrably proven in certain species of Poeciliidae that utilize nutrient exchange via placentotrophy. Conversely, superfetation has not been seen in fish of the same family that lack placentrotrophy.^5^ As humans are a placentotrophic species, superfetation is biologically plausible.

## CASE REPORT

A 31-year-old gravida 3, para 2002 presented to the emergency department with abdominal pain at 9 weeks 2 days’ gestation dated by last menstrual period (LMP), consistent with ultrasound. She conceived after taking a 5-day course of over-the-counter 50 mg clomiphene citrate, obtained abroad, on alternating days following her LMP. She reported that she used ovulation predictor kits to help time intercourse and correlated results with moliminal symptoms such as pelvic pain. ß-hCG was 169,989 mIU/mL, and transvaginal ultrasound revealed an intrauterine dichorionic diamniotic twin gestation, with fetus A measuring 9 weeks 5 days’ gestation and fetus B measuring 9 weeks 3 days’ gestation*.* The left ovary was not seen and had free fluid around it, suggesting hemorrhagic cyst rupture. A corpus luteum cyst was in the right ovary. The patient's hemoglobin was 8.9 g/dL, a substantial decrease from her prepregnancy baseline of 13 g/dL. Her abdominal examination was benign, the patient was hemodynamically stable, and she responded appropriately to pain medication. Because of her stable presentation with a nonspecific adnexal mass and visualized intrauterine pregnancy, the presumptive diagnosis of ruptured hemorrhagic cyst was made. The patient was discharged home in stable condition.

She returned 4 days later with severe abdominal pain and vaginal bleeding. Ultrasound showed a gestational sac and fetal pole with cardiac activity at 225/min in the left adnexa, consistent with an ectopic pregnancy. Crown rump length measured 2.3 cm, consistent with 9 weeks 0 days’ gestation. Complex free fluid was noted superior to the left adnexa in the Morison pouch. The twin intrauterine pregnancy was redemonstrated on this study and showed appropriate interval growth, measuring 10 weeks 1 day of gestation (Figure).

**Figure.**
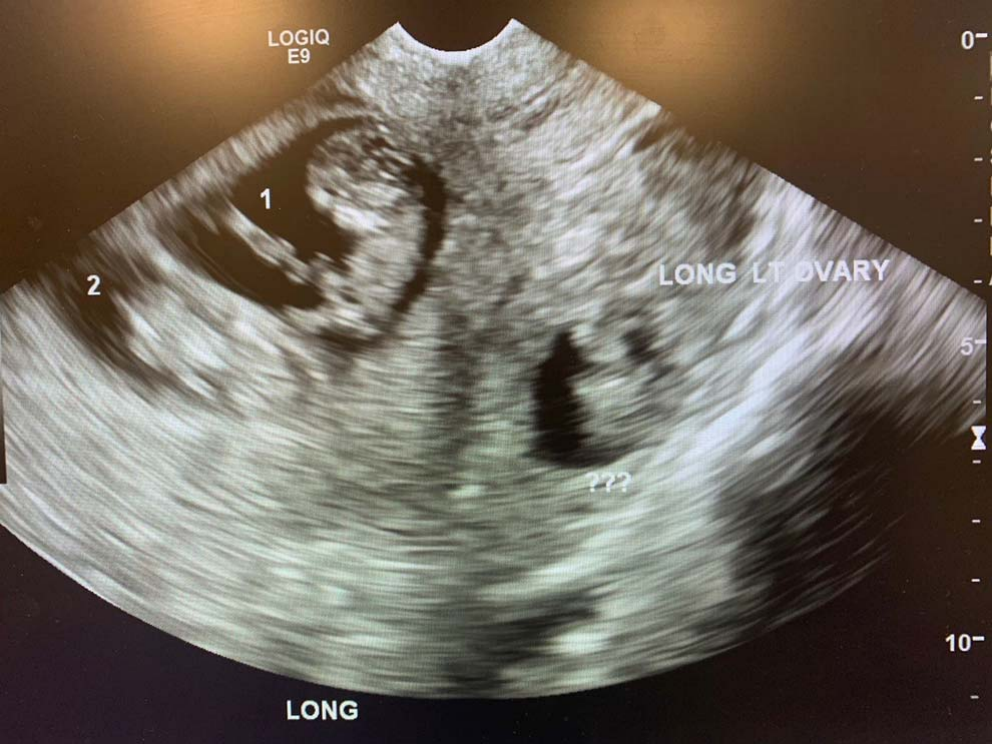
Transvaginal ultrasound view of heterotopic pregnancy.

Because of concern for ruptured ectopic pregnancy, the patient was taken to the operating room for laparoscopic salpingectomy. When the laparoscope was inserted, a ruptured left fallopian tube and approximately 1 L of hemoperitoneum were noted. Ectopic pregnancy was confirmed on tissue diagnosis.

Recovery from surgery was uneventful, and the patient was sent home postoperatively. Her pregnancy was otherwise uncomplicated, and she continued routine prenatal care until 28 weeks 4 days’ gestation when she was admitted overnight for threatened preterm labor and found to be 4 cm dilated. She was given tocolysis and betamethasone and discharged in stable condition. She presented again at 36 weeks 5 days’ gestation in preterm labor and was 9 cm dilated with malpresentation of both fetuses. She underwent primary cesarean delivery and was discharged home with healthy twins on postoperative day 2.

## DISCUSSION

Any mechanism that blocks the progression of a fertilized ovum through the fallopian tube predisposes a female to ectopic pregnancy. In this case, the proposed mechanism was the presence of an existing intrauterine pregnancy blocking the transit of the subsequently fertilized ovum. This patient released multiple oocytes capable of fertilization as a result of superovulation with clomiphene citrate. The initial 2 embryos implanted in the uterus, resulting in the dichorionic diamniotic twin gestation. The subsequent embryo was not able to migrate into the uterus and implanted in the fallopian tube. Given the difference in gestational ages between the twin gestation and the ectopic gestation, we propose that this case may be an example of human superfetation, whereby the ectopic pregnancy was fertilized after the intrauterine gestation and was unable to migrate from the fallopian tube to the uterus as a normal pregnancy would. Human superfetation has been proposed previously, but evidence is considered circumstantial, and reports are extremely rare.^4^ Nonviable pregnancies, such as those destined to miscarry and tubal ectopic pregnancies, also follow different growth trajectories than viable pregnancies.^6^

While heterotopic pregnancy is very rare, the condition has been documented in the literature, almost always in the setting of ART.^1,3,4^ This complicated condition is challenging in terms of diagnosis and treatment. The most common area of ectopic pregnancy implantation is the fallopian tube, so heterotopic pregnancies can sometimes present for the first time as a ruptured ectopic pregnancy. While the incidence of heterotopic pregnancy is estimated to be 1 in 30,000 spontaneous pregnancies, the rate increases to as high as 1 in 100 pregnancies when ART is used.^4^ Our review of the literature found that the strongest risk factor for developing a heterotopic pregnancy was ovarian hyperstimulation, which is commonly seen in females using ART. We suggest that heterotopic pregnancy should be considered in the differential diagnosis of abdominal pain for females who have undergone ovarian stimulation.

## CONCLUSION

Whether the heterotopic triplet gestation presented here resulted from the same ovulation as the intrauterine twin gestation or from a subsequent ovulation is impossible to know. Nonetheless, this report adds to the literature that perhaps superfetation can be artificially induced in humans in the presence of ART.

## Notes

*This article meets the Accreditation Council for Graduate Medical Education and the American Board of Medical Specialties Maintenance of Certification competencies for Patient Care and Medical Knowledge.*
